# Income is associated with hippocampal/amygdala and education with cingulate cortex grey matter volume

**DOI:** 10.1038/s41598-020-75809-9

**Published:** 2020-11-02

**Authors:** M. Lotze, M. Domin, C. O. Schmidt, N. Hosten, H. J. Grabe, N. Neumann

**Affiliations:** 1 Functional Imaging Unit, Center for Diagnostic Radiology and Neuroradiology, Department of Diagnostic Radiology and Neuroradiology, University Medicine Greifswald, Walther-Rathenau-Str.46, 17475 Greifswald, Germany; 2 Institute for Community Medicine, University Medicine Greifswald, Greifswald, Germany; 3 Department of Diagnostic Radiology and Neuroradiology, University Medicine Greifswald, Greifswald, Germany; 4 Department of Psychiatry and Psychotherapy, University Medicine Greifswald, Greifswald, Germany

**Keywords:** Brain, Neuroscience, Social neuroscience

## Abstract

Income and education are both elements of a person’s socioeconomic status, which is predictive of a broad range of life outcomes. The brain’s gray matter volume (GMV) is influenced by socioeconomic status and mediators related to an unhealthy life style. We here investigated two independent general population samples comprising 2838 participants (all investigated with the same MRI-scanner) with regard to the association of indicators of the socioeconomic status and gray matter volume. Voxel-based morphometry without prior hypotheses revealed that years of education were positively associated with GMV in the anterior cingulate cortex and net-equivalent income with gray matter volume in the hippocampus/amygdala region. Analyses of possible mediators (alcohol, cigarettes, body mass index (BMI), stress) revealed that the relationship between income and GMV in the hippocampus/amygdala region was partly mediated by self-reported stressors, and the association of years of education with GMV in the anterior cingulate cortex by BMI. These results corrected for whole brain effects (and therefore not restricted to certain brain areas) do now offer possibilities for more detailed hypotheses-driven approaches.

## Introduction

During the last decades, income inequality has been increasing in nearly all regions of the world and the gap between the rich and the poor is growing^1^. Both economic status (total household income or equivalent income) and education are elements of the sociologic term of socioeconomic status (SES). SES has a strong impact on physical and mental health, with higher SES going along with less chronic diseases^2^ and a longer life expectancy^3^. Thereby the brain plays an important role, because it serves as a mediator between SES and life outcomes, such as cognitive ability and emotional wellbeing^4^. At the same time, brain structure and function are influenced by SES and factors that are associated with SES. The latter include health behaviors, such as smoking, alcohol abuse, unhealthy diets and physical inactivity leading to overweight and obesity, as well as exposure to chronic stress, which tends to occur more frequently in individuals with lower SES^5^.

In a previous study, Butterworth et al.^6^ found smaller bilateral volumes of the hippocampus and amygdala in individuals who reported financial hardship during the last 12 months (n = 19) compared to those who reported no hardship (n = 384). In contrast, Gianaros et al.^7^ found that lower subjective social status, as indexed by a lower social ladder ranking, was associated with reduced gray matter volume in the perigenual area of the anterior cingulate cortex (n = 100; region of interest (ROI) analysis). Chan et al.^8^ reported a lower SES as assessed by a combination of estimated years of education and occupational socioeconomic index being associated with a reduced cortical thickness exclusively in the middle-aged, but not in the younger or elderly population. So while there is evidence of an association of SES with GMV, small sample sizes, different age groups as well as different measures for SES may have resulted in inconsistent results. Studies with a sufficiently large sample size in population based cohorts over a broad range of age and parameters of interest allowing for non-hypotheses driven analysis and sufficient spatial resolution for identification of subunits within anatomical areas are lacking. We therefore investigated if associations of the brain’s GMV in cross-sectional population cohorts are modulated by income (net equivalent income) and education (years of education). Although these parameters are associated (medium correlation of r = 0.38 in the current study), they are not interchangeable, since earnings can vary at similar educational levels. We here tested the association of equivalent income and education with GMV in 2838 adult participants (aged 21 to 90 years) derived from two population-based cohorts (SHIP2 and SHIP Trend 0). We further tested if a possible association of SES or education with GMV was mediated by alcohol, cigarettes, body mass index (BMI) or stress (burdening life events), as suggested in previous publications^4,9^.

The cohorts cover T1-weighted MRI-datasets of the whole head and questionnaires for the two parameters of interest (equivalent income, education) and possible mediators/ confounds (age, sex, alcohol and cigarette consumption, body mass index (BMI), stressors experienced over the last 12 months, total brain volume and quality of MRI). The SHIP2 cohort is a 10 years evaluation of a general population sample (SHIP0). The Trend 0 cohort represents a general population sample, too, from which those who participated in the SHIP0 cohort were excluded. Since the SHIP data were investigated with the same MRI scanner and populations did not overlap, we evaluated these cohorts together. We used CAT12 (https://www.neuro.uni-jena.de/cat/index.html#VBM) and SPM12 for VBM linear regression analyses with years of education and equivalent income as main regressors modulating GMV. For each of these analyses we inserted the following covariates: total intracranial volume (TIV), quality of images (derived from segmentation process), age, sex, body mass index, number of cigarettes (packyears), amount of alcohol (30 days). For statistical thresholding, we applied p < 0.05, family-wise error (FWE) correction for multiple comparisons for the whole brain. Additionally, we investigated possible interaction effects of education and equivalent income with age group^8^ and sex^10^. Mediation analyses were conducted with alcohol and cigarette consumption, body mass index, and self-reported stressors over the last 12 months as mediators.

## Results

### Associations of factors

When testing for associations of factors known to be associated with the brain’s GMV, we assumed a medium effect size as relevant (Cohen’s d > 0.5; for correlations r > 0.3). As expected, household income and equivalent income (household income divided by persons living in the household) were highly associated (r = 0.92; p < 0.001). Equivalent income and years of education were moderately associated (r = 0.38; p < 0.001) and therefore different linear regression analyses were performed for each. Age was weakly positively associated with equivalent income (r = 0.046, p = 0.013), but not with education (r = 0.01, p = 0.48). Women had a lower equivalent income (F_1,2836_ = 32.87, p < 0.001) and less years of education than men (F_1,2836_ = 35.95, p < 0.001) (Table 1).

**Table 1:** Characteristics of the sample grouped by sex.

	Men (mean, SD)	Women (mean, SD)	
N	1367	1471	
Age [years]	52.30 (14.1)	52.43 (13.2)	F_1,2836_ = 0.71, p = 0.79
Education [years]	12.98 (2.50)	12.44 (2.31)	F_1,2836_ = 35.95, p < 0.001
Income total [€]	2334.81 (1188.33)	2084.22 (1097.62)	F_1,2836_ = 34.10, p < 0.001
Income equiv [€]	1549.48 (754.70)	1396.34 (667.88)	F_1,2836_ = 32.87, p < 0.001
Cigarettes [packyears]	10.78 (16.30)	4.60 (9.63)	F_1,2836_ = 153.54, p < 0.001
Alcohol [g/d]	13.84 (15.72)	4.52 (6.23)	F_1,2836_ = 441.82, p < 0.001
BMI	28.12 (3.73)	27.19 (4.96)	F_1,2836_ = 31.61, p < 0.001
Stress	1.86 (0.68)	2.04 (0.77)	F_1,1795_ = 26.76, p < 0.001
TIV[ccm]	1644.91 (123.44)	1448.02 (105.12)	F_1,2836_ = 2102.2, p < 0.001
IQR	2.84 (0.34)	2.68 (0.28)	F_1,2836_ = 196.83, p < 0.001

*Edu* years of education, *Income total* total household income, *Income equiv* net-equivalent income, *Cigarettes* packyears, *Alcohol* pure alcohol in gram per day, *BMI* body mass index, *TIV* total intracranial volume, *IQR* image quality rating of the segmentation process (range: 1: very good—6: unacceptable).

### Associations of equivalent income and years of education with GMV (CAT12/SPM analyses)

**Figure 1:**
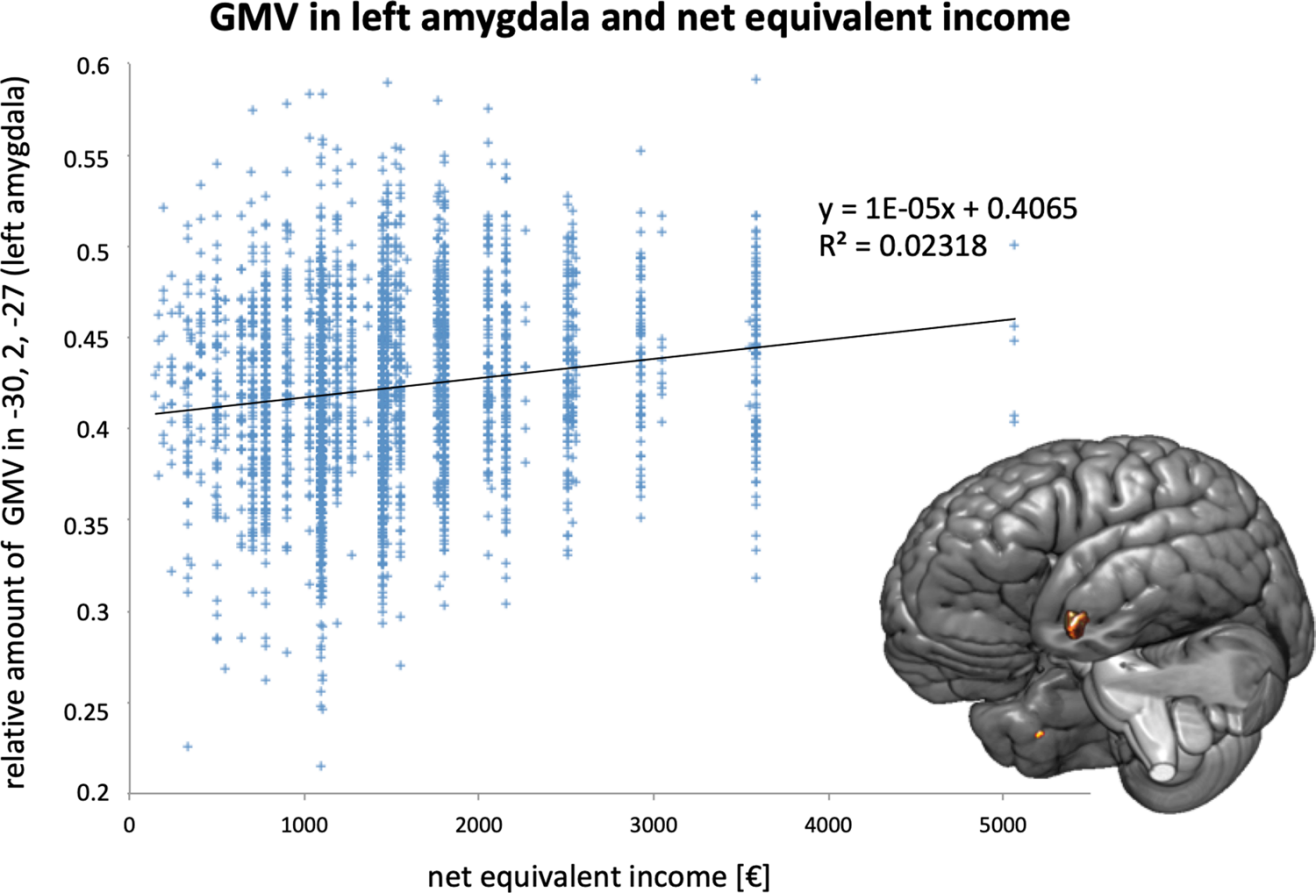
Linear regression of the relative amount of GMV at MNI coordinates [− 30, 2, − 27], left amygdala (y-axis) on equivalent income (calculated in € as the median of the range provided in the questionnaire; x-axis). The GMV effect, color coded in red/orange, is overlayed on the segmented MNI-brain.

Equivalent income was associated with GMV in the left hippocampus-amygdala complex (t = 5.55; k = 233 voxel; p_FWE_ = 0.001; MNI coordinates: − 30, 2, − 27) and the right hippocampus (t = 4.69; k = 14 voxel; p_FWE_ = 0.02; MNI coordinates: 39, − 26, − 18; see Fig. 1).

**Figure 2:**
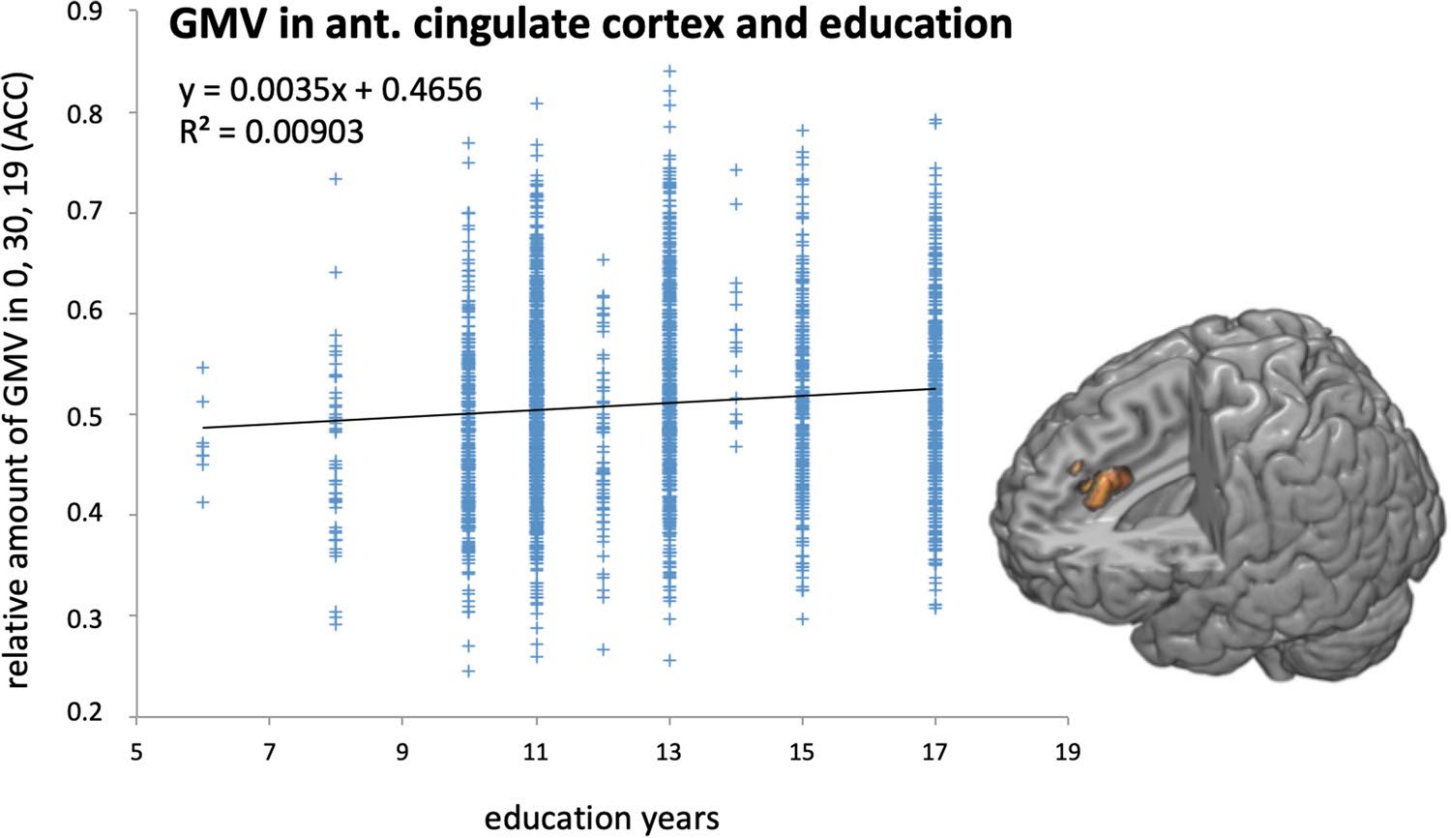
Linear regression of the relative amount of gray matter at MNI [0, 30, 20], anterior cingulate cortex/ACC (y-axis) on years of education (x-axis). Statistical map was thresholded with p_FWE_ < 0.05 and projected on a segmented T1-weighted MNI reference brain.

Years of education were associated with GMV in the bilateral anterior cingulate cortex (ACC; t = 5.02; k = 296 voxel, p_FWE_ = 0.005; MNI coordinates: 0, 30, 20; see Fig. 2).

Both effects were smaller, but still present when inserting education/equivalent income as additional regressors in the respectively other regression model. The cluster in the right hippocampus associated with equivalent income, however, did not reach the threshold of 0.05 (FWE-corrected), but was significant when using small volume correction with the right hippocampus mask (MNI: 39, − 24, − 18_;_ p_FWE_ = 0.009).

### Additional analyses with age groups

In the following, we investigated if there were interactions of equivalent income and education with age groups (whole brain FWE corrected, see Methods). The interaction of equivalent income by age group on a whole brain level was significant for the older age group in the left anterior insula (t = 4.51; k = 2 voxel; MNI: − 38, 2, 3, p_FWE_ = 0.048). The interaction of education by age group was significant for the middle early age group in the left middle temporal gyrus (t = 5.15; k = 230 voxel; MNI: − 54, − 47, 3, p_FWE_ = 0.003) and the right superior temporal gyrus (t = 4.86; k = 125 voxel; MNI: 51, − 36, 8, p_FWE_ = 0.011).

Next, we investigated if the main effects of equivalent income and education were driven by specific age sectors (ROI analysis, see “Methods”).The respective interactions of *age group by equivalent income* revealed significant voxels only in the middle late (MNI: − 33, − 3, − 23, t = 3.46, p_FWE_ = 0.004, FWE-corrected for the left hippocampus-amygdala complex and right hippocampus mask derived from the previous analysis) and older (MNI: − 30, 5, − 23, t = 3.84, p = 0.001) age groups. In the younger and middle early age group, there were no significant effects, even when using a more liberal threshold. Interaction analyses of *education by age group* showed that the main effect was present in the middle early (MNI: 0; 29; 26, t = 4.34, p = 0.001, 12; 39; 24, t = 3.82, p = 0.001, FWE-corrected for the bilateral ACC mask) and middle late (MNI: − 2; 32; 18, t = 4.32, p = 0.001, 12; 35; 15, t = 3.17, p = 0.001) age groups. In the younger and older age groups, the effect was smaller, but significant when applying small volume correction with the ACC mask^11^.

### Additional interaction analyses with sex

The interaction of *equivalent income by sex* on GMV was not significant, neither for the t-contrast males > females, nor for the t-contrast females > males after FWE correction. Applying small volume correction with a mask derived from the previous analysis (main effect of the left hippocampus-amygdala complex and right hippocampus) did not show any significant result. The identical analysis of the interaction of *education by sex* was not significant, either (small volume correction with the mask of the bilateral ACC).

### Mediation analyses

**Figure 3:**
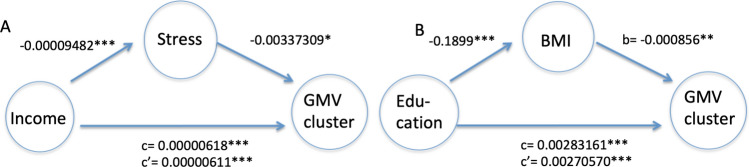
Significant mediators of the association between equivalent income and years of education (X) with the respective GMV clusters in the bilateral hippocampal/left amygdala region and the anterior cingulate cortex (Y). A/left: stressors of the last 12 months/right: body-mass index (BMI). All regression coefficients were significant as indicated with p-values.

As depicted in Fig. 3, the relationship of equivalent income with the GMV cluster in the left hippocampus-amygdala complex and right hippocampus was partly mediated by self-reported stressors (burdening life events) in the last 12 months (indirect effect ab = 0.00000032, 95% CI 0.00000004–0.00000068), and the association of years of education with the GMV cluster in the anterior cingulate cortex was partly mediated by BMI (indirect effect ab = 0.00016245, 95% CI 0.00004861–0.00029722).

## Discussion

Overall, this study identified significant associations of equivalent income and education with GMV across large cohorts without prior assumptions on regions of interest. Equivalent income was associated with left amygdala and bilateral hippocampus GMV thereby corroborating previous ROI-based findings in a smaller adult sample^6^. Hippocampal volume was also the only structure found to be associated with the individual-level socioeconomic position in a study investigating community socioeconomic disadvantage^12^. These findings are usually interpreted in the framework of a model assuming that smaller hippocampal and amygdala GMV reflect the impact of chronic stress deriving from disadvantageous life conditions^9^. Increased stress has been discussed to be a driver of increased risk for mental illness, but also circumscribed decrease of GMV^13^. We therefore used mediation analyses to test for a possible mediation between equivalent income and gray matter volume with a stress score (burdening life events during the last 12 months) as well as alcohol consumption and BMI as mediator variables. This analysis revealed a small, but significant mediation of stress for the association of equivalent income and GMV. One reason for the small effect size may be that stress was exclusively operationalized as burdening life events without taking continuous stressors deriving from adverse life conditions into account. On the other hand, the interaction of stress and poverty on GMV is complex and dependent on numerous factors, such as the timing of stress exposure. Furthermore smaller hippocampal GMV can also represent a risk factor or a marker of vulnerability for stress-related diseases rather than its consequence^14^. Further longitudinal studies in large samples are needed to disentangle these interactions, especially since stress-induced GMV decreases might diminish after years^15^. In the current study, the association of equivalent income and GMV in the bilateral hippocampal and left amygdala complex was only present in the middle late and older age groups (50–90 years). Additionally, the older age group showed an association between income and left anterior insula GMV. Possible interpretations include the protective effect of higher SES against age decline^16^ or the delayed consequences of an unhealthy lifestyle. In the current study, there may also be a specific cohort effect, since the older cohorts were born and raised in the German Democratic Republic with massive changes including widespread unemployment after its breakdown in 1989.

In contrast to previous studies (e.g.^8^), in the current study we investigated the association of education and GMV separately from equivalent income. Our data of an association of the GMV of the anterior cingulate cortex with education corroborate findings reported in a previous study in an elderly sample^17^. GMV in the ACC mediates the association between family SES and a depression-related trait^18^ underlining the role of this area for emotional control. The anterior cingulate cortex has also been linked to learning^19^ and higher control of reward-related regions—the ability to accept delayed but higher financial reward after longer education might well be related to that structural specification. Further, there was an additional association between education and GMV in the bilateral superior and middle temporal gyri (encompassing Wernicke’s area and its right-hemispheric analogue) only in the middle early age group (35–49 years), what may underline the importance of language processing in higher education. We here demonstrated that the duration of education and equivalent income should be considered separately in adults, because they are associated to the GMV of different structures. Further, the association of education with GMV in the ACC was partly mediated by BMI what corroborates findings of age-independent influences of BMI on gray matter (as well as its reverse effect^20^) in the ACC^21^.

With respect to the meta-analysis of Yaple and Yu^22^ differences in findings might be based on (A) the different evaluation methods applied (VBM-analysis of all data in our approach and effect size–seed-based d Mapping applied by Yaple and Yu); and (B) different age ranges of the samples investigated. However, smaller bilateral hippocampus GMV was also associated with low SES in their meta-analysis, which is underlined by our study.

In conclusion our cohort study supports previous assumptions on interactions of brain structure and SES raised by Farah^4^. Associations of low SES with brain structure are especially important, because they might provide a basis of generation overlapping effects of prefrontal executive control function, reward dependency of behavior and resilience to the effects of external stressors increasing the risk for psychopathology.

## Material and methods

### Participants

We investigated two general-population samples from the Study of Health in Pomerania (SHIP^23^). These comprised data from SHIP2 and Trend0 (data collection 2008–2012); together 2838 participants; 1367 men, mean age 52.37 ± 13.64 years (range 21–90). The study protocol was approved by the Ethics Committee of the University Medicine of Greifswald and written informed consent was obtained from each subject. In addition, all methods were performed in accordance with the relevant guidelines and regulations.

### MRI assessments

All brain images were obtained with the same 1.5 T Siemens MRI scanner (Magnetom Avanto, Siemens Medical Systems, Erlangen, Germany) without software updates during the evaluation period. More specifically a T1-weighted magnetization prepared rapid acquisition gradient echo (MPRAGE) sequence was used with the following parameters: 176 slices, matrix = 256 × 176 pixels, voxel size = 1.0 mm isotropic, slice thickness = 1.0 mm, repetition time = 1900 ms, echo time = 3.37 ms, flip angle 15°. These details have also been provided in different other manuscripts on this cohort before^23^.

### Assessment of variables

Paper and pen questionnaires were provided to the participants for the following variables: Total household equivalent income: equivalent income available per month (1: < 500, 2: 500–900, 3: 900–1300, 4: 1300–1800, 5: 1800–2300, 6: 2300–2800, 7: 2800–3300; 8: 3300–3800). If no answer was provided, the participant was excluded. Questions: Persons living in the household (0, 1, 2, 3, 4, 5 etc.). Net equivalent income was calculated as the total household equivalent income divided by number of persons in household. Education was assessed in absolute years.

### Quality control and exclusion of pathologies

All MRI head scans were visually inspected with regard to image artifacts and clinical abnormalities. Any brain images indicating stroke, multiple sclerosis, epilepsy, Parkinson’s disease, dementia, cerebral tumor, intracranial cyst or hydrocephalus were excluded, leaving 1081 (SHIP-2) and 2046 (SHIP-Trend-0) images. Furthermore, subjects with recorded intake of anxiolytics or opioids, as well as with PHQ9 (Patient Health Questionnaire with 9 responses) depression scores^24^ greater than 14 were excluded, leaving 1,037 (SHIP-2) and 1984 (SHIP-Trend-0) images. Finally, all subjects with incomplete datasets for possible confounds (i.e., age, years of education, nicotine intake, alcohol consumption, body mass index) were excluded. The final sample contained 2,838 subjects, with 967 subjects from SHIP-2 and 1,871 subjects from SHIP-Trend-0. The method used here has a high overlap with those described before in more detail^10^.

### Data analyses

T1-weighted images were preprocessed in MATLAB (The MathWorks, Natick, MA) using Statistical Parametric Mapping, version 12 (SPM12; Wellcome Department of Cognitive Neurology, University of London) and the Computation Anatomy Toolbox (CAT) for SPM (CAT 12; Christian Gaser; Department of Psychiatry, University of Jena) applying CAT12 default parameters. Images were corrected for magnetic field inhomogenities, spatially normalized using the DARTEL algorithm^25^, and segmented into GM, white matter (WM), and cerebrospinal fluid (CSF). The segmentation process was further enhanced by accounting for partial volume effects and by using a hidden Markov Random Field (MRF) model. Finally, the resulting GM segments were smoothed using a Gaussian kernel of 8 mm full width at half maximum (FWHM). Quality of images was assessed by using the automated image quality rating (IQR) included in the CAT12 toolbox (https://www.neuro.uni-jena.de/cat/index.html#VBM). It constitutes a weighted average of the local (noise contrast ratio) and global (inhomogeneity contrast ratio) standard deviations within the optimized white matter segment scaled by the minimum tissue contrast, and the root mean square of the voxel size. The obtained quality ratings range from 0.5 (100 rating points) to 10.5 (0 rating points) with values around 1 and 2 describing (very) good image quality (grad A and B) and values around 5 (grad E) and higher (grad F, < 50 rating points) indicating problematic images. However, excellent rating is defined for extraordinary good images that were measured on high field systems, whereas typical scientific (clinical) data is expected to get just good to satisfactory ratings. Our current sample had a mean IQR value of 2.76 (s = 0.32, range 2.19−4.71). Total brain volume (TBV) was calculated as sum of GM, WM, and CSF.

### Statistical analyses

Linear regression analyses were conducted for the variables equivalent income and years of education. As covariates the following factors were inserted: total intracranial volume (TIV), IQR, age, sex, BMI, number of cigarettes (packyears), amount of alcohol (30d).

For statistical thresholding, we applied p < 0.05, family-wise error (FWE) corrected for multiple comparisons for the whole brain. Spatial assignment of effects was conducted with the SPM Anatomy Toolbox Version 2.2c (for the amygdala^26^), for further subdivison of the hippocampus we used those suggested by Plachti et al.^27^. If cytoarchitectural differentiation was not available, with the Automated Anatomical Labeling Toolbox (AAL; for the ACC^28^). Brain areas were superimposed on the MNI render brain and on the T1-weighted Collins single-participant brain.

### Full-factorial model with age and sex

Age groups were defined according to Chan et al.^8^ (younger : 21–34 years, n = 300; middle early: 35–49 years, n = 925; middle late: 50–64 years, n = 986; older: 65–90 years, n = 627). We calculated the respective interactions in a full-factorial model including age group (4 levels: see above) and sex (2 levels; male, female) as factors, and equivalent income or education as regressors. Nuisance variables were the same as before (cigarettes, alcohol, BMI, total intracranial volume, image quality rating). Interactions with age and sex were investigated in a whole brain analysis (FWE corrected) and with regard to the main effects of equivalent income and education on GMV from the previous analysis. For the latter, we used small volume correction (FWE-corrected) for the left hippocampus-amygdala complex/right hippocampus and ACC, respectively.

### Mediation analysis

We performed a mediation analysis (PROCESS macro^29^ v. 3.5, for SPSS, model 4, 10,000 bootstrap samples) for the association of equivalent income and education with the correspondent gray-matter clusters (relative amount of GMV averaged over the clusters of significance derived from the whole brain VBM). Mediators comprised the consumption of alcohol and cigarettes, BMI, and self-reported stressors of the last 12 months. The latter was only available for the T0 cohort of 1984 participants. Since equivalent income and years of education were differently associated with assumed mediators (Table 2), we tested alcohol, BMI, and stress as mediators in the income mediation analysis, and alcohol, cigarettes, and BMI as mediators for the education mediation analysis. Age, sex, TIV, and IQR were inserted as covariates. Stressors were assessed using a subscale of the Questionnaire for the Assessment of Health Behaviour^30^ comprising eight questions: Were there stressing/burdening events in the following areas of life during the last 12 months: work/school/career; marriage/partnership; family/children; friends; leisure time; financial situation; housing situation; health. Questions were answered on a Likert scale ranging from 1–5 (1: no events, 5: very incriminatory events) and were averaged to one parameter.

**Table 2:** Health behaviour in different age groups and correlations with equivalent income and years of education.

	All ages	Younger (21–34 y) (mean, SD)	Middle early (35–49 y) (mean, SD)	Middle late (50–64 y) (mean, SD)	Older (65–90 y) (mean, SD)
N	2832	300	925	986	627
Alcohol Corr Income Corr Education	9.01 (12.68) r = 0.102** r = 0.090**	7.51 (11.02) r = − 0.046 r = − 0.016	9.71 (14.12) r = 0.051 r = − 0.038	9.96 (13.08) r = 0.141** r = 0.122**	7.19 (10.02) r = 0.131** r = 0.262**
Cigarettes Corr Income Corr Education	7.58 (13.62) r = − 0.019 r = − 0.064**	3.54 (5.65) r = − 0.121* r = − 0.248**	7.49 (11.24) r = − 0.070* r = − 0.204**	8.88 (15.60) r = − 0.025 r = − 0.077*	7.59 (15.69) r = 0.070 r = 0.033
BMI Corr Income Corr Education	27.63 (4.43) r = − 0.052** r = − 0.099**	25.32 (4.28) r = 0.002 r = − 0.108	26.72 (4.17) r = − 0.012 r = − 0.113**	28.60 (4.48) r = − 0.120** r = − 0.113**	28.57 (4.13) r = − 0.031 r = − 0.119**
Stress: N = 1797 Corr Income Corr Education	1.96 (0.73) r = − 0.111** r = − 0.043	2.17 (0.75) r = − 0.101 r = − 0.078	2.08 (0.73) r = − 0.103* r = − 0.051	1.89 (0.72) r = − 0.124** r = − 0.006	1.69 (0.65) r = − 0.197** r = − 0.046
Income equiv	1470.11 (715.00)	1311.48 (721.0)	1551.43 (733.1)	1560.62 (789.2)	1283.69 (475,1)
Education	12.70 (2.42)	12.55 (1.91)	12.61 (2.17)	12.96 (2.50)	12.50 (2.80)

*Alcohol* pure alcohol [g/d], Cigarettes [packyears]; *BMI* body mass index, *Stress* burdening events during the last 12 months, *Income equiv* net equivalent income; education in years; *corr* Pearson correlation; *p < 0.05, **p < 0.01.
